# Multicenter Prevalence Study Comparing Molecular and Toxin Assays for *Clostridioides difficile* Surveillance, Switzerland

**DOI:** 10.3201/eid2610.190804

**Published:** 2020-10

**Authors:** Andreas F. Widmer, Reno Frei, Ed J. Kuijper, Mark H. Wilcox, Ruth Schindler, Violeta Spaniol, Daniel Goldenberger, Adrian Egli, Sarah Tschudin-Sutter

**Affiliations:** University Hospital Basel, Basel, Switzerland (A.F. Widmer, R. Frei, R. Schindler, V. Spaniol, D. Goldenberger, A. Egli, S. Tschudin-Sutter);; Leiden University Medical Center, Leiden, the Netherlands (E.J. Kuijper);; Leeds Institute of Biomedical and Clinical Sciences, University of Leeds, and Leeds Teaching Hospitals, Leeds, UK (M.H. Wilcox)

**Keywords:** Clostridioides difficile, antimicrobial resistance, healthcare-associated infections, enteric infections, bacteria, molecular assay, toxin assay, surveillance, diagnosis, Switzerland

## Abstract

Public health authorities in the United States and Europe recommend surveillance for *Clostridioides difficile* infections among hospitalized patients, but differing diagnostic algorithms can hamper comparisons between institutions and countries. We compared surveillance based on detection of *C. difficile* by PCR or enzyme immunoassay (EIA) in a nationwide *C. difficile* prevalence study in Switzerland. We included all routinely collected stool samples from hospitalized patients with diarrhea in 76 hospitals in Switzerland on 2 days, 1 in winter and 1 in summer, in 2015. EIA *C. difficile* detection rates were 6.4 cases/10,000 patient bed-days in winter and 5.7 cases/10,000 patient bed-days in summer. PCR detection rates were 11.4 cases/10,000 patient bed-days in winter and 7.1 cases/10,000 patient bed-days in summer. We found PCR used alone increased reported *C. difficile* prevalence rates by <80% compared with a 2-stage EIA-based algorithm.

Since its identification as a cause of antibiotic-associated pseudomembraneous colitis in 1978 (*1*), *Clostridioides difficile* has emerged as a major healthcare-associated pathogen worldwide. In the United States, *C. difficile* infection (CDI) rates doubled during 1996–2003 (*2*), and rates of CDI were reported to be 76.9 cases/10,000 discharges in 2005 (*3*). In a more recent national point-prevalence study including US healthcare facility in-patients, 13/1,000 patients were found to be either infected or colonized (*4*), a higher rate than had been previously estimated. In a national point-prevalence study of nosocomial infections in the United States, *C. difficile* was the most common causative pathogen overall (*5*). The increase largely has been attributed to the emergence of the hypervirulent strain, PCR ribotype 027 (RT027), which was identified as causative strain in 82% of cases during CDI outbreaks in Quebec, Canada, during 2001–2003 and accounted for 31% of all cases of healthcare-associated infections in the United States in 2011 (*6*–*9*). In Europe, CDI incidence varies across hospitals and countries with a weighted mean of 4.1 cases/10,000 patient-days per hospital in 2008 (*10*). The most recent study on CDI prevalence in Europe suggests an increase in the number of cases, reporting a mean of 7.0 cases/10,000 patient-bed days and ranging among countries from 0.7 to 28.7 cases/10,000 patient-bed days (*11*). The most common ribotype identified was RT027, which was detected in 4 countries: Germany, Hungary, Poland, and Romania (*11*).

To estimate and compare the burden of CDI across the United States, the US Centers for Disease Control and Prevention (CDC) began population-based CDI surveillance in 10 locations in 2011 (*12*). The European Centre for Disease Prevention and Control (ECDC) began coordinating CDI surveillance in acute care hospitals in Europe in 2016 (*13*). Both authorities provide case definitions based on different diagnostic approaches, including detection of *C. difficile* toxin A and B by enzyme immunoassay (EIA) or detection of toxin-producing *C. difficile* organisms by PCR. However, the use of different diagnostic algorithms to detect *C. difficile* might hamper comparisons between institutions and countries. Therefore, in a nationwide *C. difficile* multicenter prevalence study in Switzerland, we systematically compared surveillance measures based on detection of *C. difficile* in stool by either PCR as a stand-alone test or by a 2-stage algorithm consisting of an EIA to detect glutamate dehydrogenase (GDH) and toxins A and B.

## Methods

### Study Design

We performed a nationwide multicenter prevalence study of toxigenic *C. difficile* detected in stool samples routinely collected from hospitalized patients with diarrhea. Our study followed the design of a previous point-prevalence study for maximal comparibility between our results and data from Europe (*11*). University Hospital Basel, a tertiary care center in Switzerland, coordinated the study. All hospitals participating in Swissnoso (https://www.swissnoso.ch), a national infection prevention network, were asked to participate. The Swissnoso network consists of 85 acute care hospitals that account for a total of 26,341 beds.

The Ethics Committee Northwest and Central Switzerland (Ethikkommission Nordwest-und Zentralschweiz) issued a declaration of no objection for this study. We adhered to STROBE guidelines for reporting on observational studies (*14*).

### Sample Collection

All stool samples collected from inpatients >1 year of age with diarrhea that were submitted to the microbiology laboratory on 2 specified sampling days, 1 in winter and 1 in summer, in 2015 were eligble for inclusion. Only 1 sample per patient was included. In addition, stool samples that tested positive for toxigenic *C. difficile* <1 week prior to each study day also were collected from all institutions to obtain a more detailed estimate of ribotype distribution in Switzerland.

We collected the following institutional data for all hospitals and their affiliated microbiology laboratories: contact information; detailed information regarding laboratory diagnostics in place; and detailed information on the total number of admissions, number of beds, and number of patients hospitalized on the 2 days of the study. We also collected information on the total number of diagnosed CDI cases at each institution during the study year. For each eligible stool sample, we collected the following data: date of sample collection, age and gender of patient, ward location and clinical specialty, institution, whether a *C. difficile* test was ordered by the treating physician, and result of the *C. difficile* test if testing was performed at the local laboratory.

### Procedures

We tested all stool samples at the Division of Clinical Microbiology of the University Hospital Basel by using a 2-stage algorithm consisting of EIA and PCR. We performed EIA to detect GDH and toxins A and B by using C. DIFF QUIK CHEK COMPLETE (Techlab, https://www.techlab.com), following the manufacturer’s instructions. We then performed PCR to detect the toxin B gene by using the RealStar PCR Kit (Altona Diagnostics, https://www.altona-diagnostics.com). For detected *C. difficile*, we performed strain typing by using high-resolution capillary gel-based PCR ribotyping according to the method previously described by Stubbs et al. (*15*).

### Outcomes

We calculated reported and measured rates of detected toxigenic *C. difficile* per 10,000 patient bed-days across participating institutions. We compared differences in testing algorithms for detection of toxigenic *C. difficile* across institutions in Switzerland and performance characteristics of diagnostic algorithms. We considered the proportion of missed toxigenic *C. difficile* cases and ribotype distributions as additional outcomes. We further assessed the proportion of laboratories using optimized *C. difficile* diagnostic tests, which we defined as using an algorithm recommended by the European Society of Clinical Microbiology and Infectious Diseases (*16*).

### Statistical Analyses

We separately calculated rates for each diagnostic algorithm performed in the coordinating center laboratory. In addition, we separately calculated rates for dedicated children’s hospitals. We defined missed *C. difficile* cases as those in which tests were negative at the participating hospital’s laboratory but positive at our institiution. We used descriptive statistics to report ribotypes and differences in diagnostic algorithms across all participating institutions. All analyses were perfomed in Stata version 15.1 (StataCorp, https://www.stata.com).

## Results

**Figure 1:**
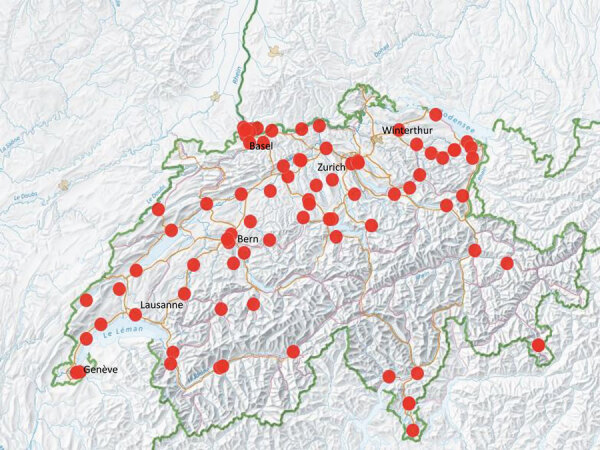
Distribution of centers participating in a prevalence study comparing molecular and toxin assays for nationwide surveillance of *Clostridioides difficile*, Switzerland. Red circles represent location of participating centers.

Participating institutions included 76/85 (89.4%) institutions belonging to the Swissnoso network. Among participating institutions, 5 were academic teaching hospitals, 3 were dedicated children’s hospitals, and 36 were affiliated microbiology laboratories. Participating institutions were distributed across all geographic regions of Switzerland (Figure 1).

Participating institutions reported collecting a fecal sample for microbiological workup in »65% (SD +25%) of all patients with hospital-onset diarrhea. Among participating institutions, 15/76 (19.7%) did not begin CDI treatment before fecal sample collection. Among institutions that intitiated treatment before collecting fecal samples, 23/76 (30.3%) began treatment in <2% of patients, 12/76 (15.8%) began treatment in 3%–5% of patients, 8/76 (10.5%) began treatment in 6%–10% of patients, and 1 (1.3%) began treatment in 11%–20% of patients. The other 17 (22%) institutions were not able to provide an estimate of these data. 

Overall, 354 stool samples were submitted to the coordinating center, of which 338 were eligible for study inclusion; 16 samples were excluded because they were not liquid, their submitted data were incomplete, or they were duplicate samples from 1 patient. Among 338 samples included, 250 were collected as part of the point-prevalence study. We excluded 8 of these because the samples were collected from patients <1 year of age. In all, we included 242 samples in the point-prevalence study.

### Diagnostic Algorithms

**Figure 2:**
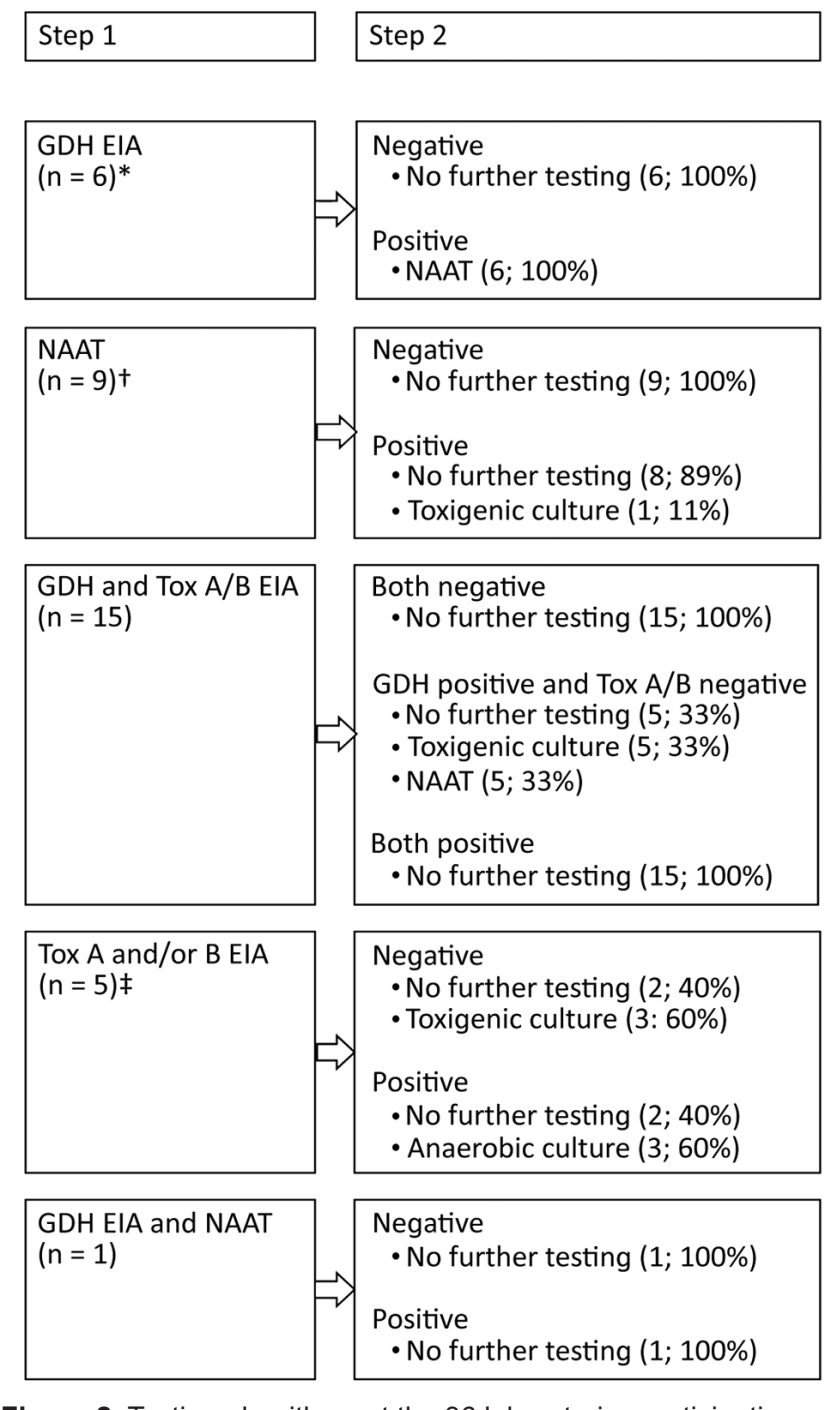
Testing algorithms at the 36 laboratories participating in a prevalence study comparing molecular and toxin assays for nationwide surveillance of *Clostridioides difficile*, Switzerland. EIA, enzyme immunoassay; GDH, glutamate dehydrogenase; NAAT, nucleic acid amplification test; Tox, toxin. *Seven samples taken during the summer sampling period. †Ten samples taken during the summer sampling period. ‡Three samples taken during the summer sampling period.

Among the 36 participating laboratories, 1 routinely tested all diarrheal stool samples for toxigenic *C. difficile* and 35 tested only if a specific test was requested. Optimized diagnostic tests for detection of toxigenic *C. difficile* were used by 58% (21/36) of laboratories in the winter sampling period and by 61% (22/36) in the summer sampling period. Among laboratories not following the recommendations of the European Society of Clinical Microbiology and Infectious Diseases (*16*), 9 in the winter sampling period and 10 in the summer sampling period used a nucleic acid amplification test (NAAT) alone, and 5 in the winter sampling period and 3 in the summer sampling period used EIAs for A and B toxins either as a standalone test or as an initial screening test. Only 1 laboratory reported having established PCR ribotyping methodologies (Figure 2). 

### Point-Prevalence Analyses

We collected demographic characteristics of patients whose stool samples tested positive by our testing algorithms (Table 1). *C. difficile* tests were required and performed for 68% (165/242) of stool samples; 6% (27/165) were reported as positive by the affiliated microbiology laboratory.

**Table 1:** Demographic data for 242 patients whose stool samples were included in the study of detection of *Clostridioides difficile* via PCR and enzyme immunoassay for glutamate dehydrogenase and A and B toxins, Switzerland*

Demographics	All patients	Method of *Clostridioides difficile* detection	
		EIA for GDH and A and B toxins, n = 21	PCR, n = 30
Median age, y (IQR)	63 (44–80)	79 (59–86)	78 (55–85)
Sex			
M	104 (43.0)	6 (28.6)	10 (33.3)
F	131 (54.1)	15 (71.4)	20 (66.7)
Not reported	7 (2.9)	0	0
Clinical specialty			
Medical	127 (52.5)	11 (52.4)	11 (36.7)
Surgery	43 (17.8)	3 (14.3)	6 (20.0)
Obstetrics, gynocology	3 (1.2)	0	0
Pediatrics	21 (8.7)	1 (4.8)	3 (10.0)
Other	28 (11.6)	5 (23.8)	7 (23.3)
Not reported	20 (8.3)	1 (4.8)	3 (10.0)
Intensive care	40 (16.5)	5 (23.8)	5 (16.7)

*Values are reported as no. (%) except where indicated. EIA, enzyme immunoassay; GDH, glutamate dehydrogenase; IQR, Interquartile range.

At the coordinating center, we detected *C. difficile* in 9% (21/242) of samples by EIA for GDH and A and B toxins and in 12% (30/242) of samples by PCR alone. Among all 27 samples reported as positive by the participating centers, we confirmed 18 (67%) by EIA and 24 (89%) by PCR. Among 138 samples reported as negative by the participating centers, 1 (1%) sample tested positive by EIA and 3 (2%) tested positive by PCR at the coordinating center. Among 77 samples not tested for *C. difficile* at the participating centers, we detected *C. difficile* in 2 (3%) by EIA and in 3 (4%) by PCR. Among 21 stool samples that tested positive by EIA at the coordinating center, a *C. difficile* test was not requested in 2 (10%) cases. Among 30 samples that tested positive by PCR at the coordinating center, a *C. difficile* test was not requested in 3 cases (10%; Table 2).

**Table 2:** Underdiagnosis and misdiagnosis of *Clostridioides difficile* infection at participating hospitals, Switzerland*

Method of detection	No. samples submitted	No. samples tested	Undiagnosed, no. (% of all positive samples)	False-positive, no. (%)	False-negative, no. (%)
EIA for GDH and A and B toxins	242	165	2 (9.5)	9 (5.5)	1 (0.6)
PCR	242	165	3 (10)	3 (1.8)	3 (1.8)

*EIA, enzyme immunoassay; GDH, glutamate dehydrogenase.

Measured detection and testing rates of toxigenic *C. difficile* were higher than the reported rates across all participating institutions (Table 3). Depending on the diagnostic algorithm applied, the largest difference in prevalence across all institutions was measured during the winter sampling period, which had a prevalence of 6.4 cases/10,000 patient bed-days by EIA and 11.4 cases/10,000 patient bed-days by PCR alone. Thus, across all institutions, rates of toxigenic *C. difficile* detection by PCR alone were <80% higher than detection rates by EIA for GDH and A and B toxins. In addition, detection rates by PCR alone were <100% higher in dedicated children’s hospitals (Table 3).

**Table 3:** Reported and measured detection and testing rates of toxigenic *Clostridioides difficile*, Switzerland, 2015*

Institutions and testing methods	Reported rate/10,000 patient bed-days	Measured rate/10,000 patient bed-days, winter (range)	Measured rate/10,000 patient bed-days, summer (range)	Mean measured rate/10,000 patient bed-days (range)	Testing rate/10,000 patient bed-days (range)
All institutions	3.8 (0–11)				67.5 (0–3,202)
EIA		6.4 (0–387)	5.7 (0–475)	6.1 (0–475)	
NAAT		11.4 (0–387)	7.1 (0–475)	9.3 (0–475)	
Children’s hospitals	1.1 (0.4–1.1)				22.5 (7.0–46.7)
EIA		33.7 (0–73)	0	16.9 (0–73)	
NAAT		67.3 (0–99)	0	33.6 (0–99)	

EIA, enzyme immuno assay; GDH, glutamate dehydrogenase; NAAT, nucleic acid amplification tests.

### Ribotype Distribution

**Figure 3:**
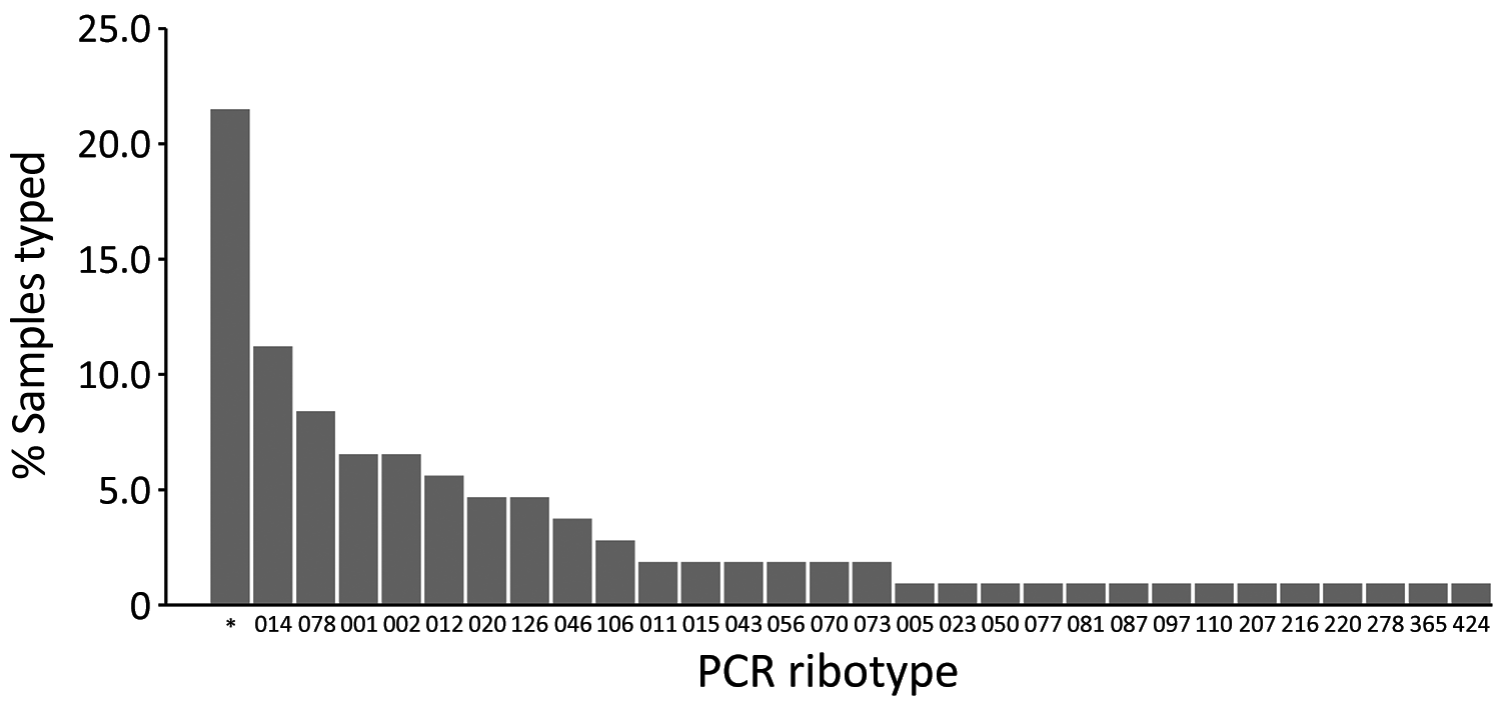
Distribution of PCR ribotypes among 107 samples collected in a prevalence study comparing molecular and toxin assays for nationwide surveillance of *Clostridioides difficile*, Switzerland. *Unknown ribotype.

We cultured and ribotyped 107 toxigenic *C. difficile* strains, 29 from the 2 point-prevalence days and 78 collected <1 week before each prevalence day. We identified a large diversity of *C. difficile* ribotypes, 23 (22%) had not been referenced before. The ribotypes most commonly identified included RT014 (12/107; 11%), presumably hypervirulent RT078 (9/107; 8%), RT001 (7/107; 7%), and RT002 (7/107; 7%) (Figure 3).

## Discussion

In this nationwide multicenter study, we found that PCR as a stand-alone test increased reported *C. difficile* prevalence rates <80% compared with a 2-stage EIA-based algorithm. At first glance, this finding was not surprising given the higher sensitivity of EIA (*16*). However, the fact that our results and conclusions are based on a nationwide cohort representing all geographic regions of Switzerland adds to the study’s credibility. In addition, our results strengthen the advice of the European Society of Clinical Microbiology and Infectious Diseases study group for *C. difficile* against use of a single commercial test for diagnosing CDI because of the low positive predictive values when CDI prevalence is low, 46% at a CDI prevalence of 5% (*16*). However, CDC and ECDC protocols for CDI surveillance define a case of CDI as the combination of diarrheal stool and a positive PCR (*12*,*13*). In addition, the clinical practice guidelines for CDI in adults and children published by the Infectious Diseases Society of America and Society for Healthcare Epidemiology of America recommend testing by different approaches, such as multistep algorithms or NAAT, depending on the degree of clinical suspicion (*17*). Based on a systematic review and meta-analysis, the American Society of Microbiology also recommends different approaches, including NAAT-only testing, and algorithms that include GDH and NAAT or GDH, toxins, and NAAT (*18*). Although these recommendations stand to reason for detection of CDI in individual patients, our results challenge their utility for meaningful comparisons in surveillance studies and suggest that uniform definitions should be provided.

On both point-prevalence days, we noted a higher nationwide rate of toxigenic *C. difficile* than previously reported in incidence studies performed at different institutions in Switzerland (*19*–*21*). Our findings suggest that CDI rates have increased during the last decade in Switzerland, a finding that is in line with reports from other countries in Europe (*11*). Using the same diagnostic algorithm, diagnostic test, and a similar study design to the multicenter point-prevalence study of CDI in hospitalized patients with diarrhea in Europe, the nationwide mean prevalence rates are comparable in Switzerland (mean 6.1 cases/10,000 patient bed-days) and Europe (7.0 cases/10,000 patient bed-days) (*11*). Because we only included liquid stools in our study, our mean prevalence rate of 9.3 cases/10,000 patient bed-days measured by PCR fulfills the ECDC case definition and further shows that CDI is increasing substantially nationwide.

We found a lower proportion of missed detection of toxigenic *C. difficile* in Switzerland (9.5%), driven by the absence of clinical suspicion, compared with Europe (23%), which equates to 1 missed case of *C. difficile* per day among the included institutions in Switzerland. False-negative testing accounted for 1 additional missed diagnosis during both point-prevalence days, which extrapolates to »550 missed cases of *C. difficile* per year among hospitals across the nation.

We detected a variety of different RTs during our study period, 21% of which had not been referenced before. Of note, we did not recover hypervirulent RT027, but RT078 was the third most common strain circulating in Switzerland during our study. In contrast, a point-prevalence study in Europe identified RT027 as the most commonly circulating strain during its study period but did not detect RT078. RT078 has been associated with similarly severe disease manifestations as RT027, but RT078 has been reported to affect younger patients and to be linked more commonly with community-associated disease in the Netherlands (*22*). RT078 has been isolated from piglets with diarrhea, possibly suggesting ongoing transmission by introduction to the food chain because isolates from humans and pigs were found to be highly genetically related (*22*). A component of RT078 infections also was reported in Northern Ireland, which has a large pig population and »1:1 ratio of cattle to humans (*23*). In Switzerland, RT078 has been isolated previously from 6 wastewater treatment plants, suggesting its dissemination in the community (*24*). Except for both hypervirulent RT027 and RT078, we identified other similarities in RT distribution between Switzerland and the rest of Europe; RT014, RT001, RT002, and RT020 were among the 10 most commonly identified ribotypes in both settings.

Our study has some limitations, most of which are intrinsic to point-prevalence studies. First, our study only reflects frequency of toxigenic *C. difficile* detected on 2 days in 2015; therefore, we cannot draw solid conclusions regarding incidence. We expanded the timeframe for assessing the distribution of ribotypes circulating in Switzerland by an additional week for each prevalence day, but this still represents a limited collection of the true incidence. Second, we cannot rule out introduction of bias to testing policies at the participating hospitals, which might have increased testing on the 2 point-prevalence days. However, we did not provide any promotional information regarding our study, so alterations in daily clinical practice among treating physicians is unlikely on these 2 days. Third, we only included liquid stool samples for analyses, but we did not consider any other preanalytical factors, such as the use of laxatives, for testing eligibility. Finally, we applied surveillance definitions recommended by CDC and ECDC rather than defintions used for the clinical diagnosis of CDI in individual patients, such as detection of *C. difficile* in the context of symptoms related to CDI. Therefore, we cannot rule out detection of toxigenic *C. difficile* from colonization rather than infection in some cases.

In conclusion, this nationwide multicenter study reveals that PCR as a stand-alone test results in an increase of *C. difficile* prevalence rates of <80% compared with a 2-stage EIA-based algorithm. Our findings underscore the need for consistent testing algorithms for meaningful interinstitutional and nationwide comparisons. Our results also challenge the utility of the current CDC and ECDC case definitions and highlight the need for uniform recommendations on diagnostic approaches. 

Appendix List of hospitals and laboratories participating in a point-prevelance study on *Clostridioides difficile* surveillance, Switzerland.
