# Supplementary material for: Multicenter Prevalence Study Comparing Molecular and Toxin Assays for Clostridioides difficile Surveillance, Switzerland
Source: Emerg Infect Dis. 2020 Oct;26(10):2370–7. doi: 10.3201/eid2610.190804 (PMC7510716; doi:10.3201/eid2610.190804)
Supplement: Appendix — List of hospitals and laboratories participating in a point-prevelance study on Clostridioides difficile surveillance, Switzerland. [file 19-0804-Techapp-s1.pdf]

Article DOI: https://doi.org/10.3201/eid2610.190804

Multicenter Prevelance Study Comparing Molecular and Toxin Assays for *Clostridioides difficile* Surveillance, Swtizerland

Appendix

Acknowledgments

We thank all participating centers and local coordinators (in alphabetical order): Mauro Albertini, Casa Anziano Poschiavo centre # 12401A und Ospedale San Sisto, Reshma Autar, Kantonsspital Baden AG, Carlo Balmelli, Ente Ospedaliero Cantonale (EOC) Ospedale Regionale di Lugano – Civico und Ospedale Regionale di Bellinzona – San Giovanni und Ospedale Regionale di Locarno – La Carità und Ospedale Regionale di Mendrisio – Beata Vergine, Christoph Berger, Universitäts-Kinderkliniken Zürich – Eleonorenstiftung, Marianne Blatter, Spital Netz Bern AG Spital Münsingen und Spital Ziegler und Spital Tiefenau und Spital Riggisberg, Thomas Bregenzer, Spital Lachen AG, Olivier Clerc, Hôpital Neuchâtelois HNE Site de la Chaux-de-Fonds und de Pourtalès, Christian Chuard, Hôpital fribourgeois HFR Hôpital cantonal – Site de Fribourg, Alain Cometta, Etablissements Hospitaliers du Nord Vaudois eHnv – Site d'Yverdon, Thanh Dang, Clinique des Grangettes, Mirjam de Roche, Spital STS AG, Spital Zweisimmen, Stefan Drechsel, Spital Davos, Martin Egger, Regionalspital Emmental Burgdorf und Langnau, Gerhard Eich, Stadtspital Triemli, Katja Eigenmann, Zuger Kantonsspital AG, Felix Fleisch, Kantonsspital Graubünden, Ursula Maria Flückiger, Hirslanden Klinik Aarau, Manfred Füeg, Merian Iselin Klinik für Orthopädie und Chirurgie, Urs Führer, Spitalzentrum Biel AG, Christoph Fux, Kantonsspital Aarau und Asana Gruppe AG Spital Leuggern und Menziken, Peter Graber, Kantonsspital Baselland Liestal, Andreas Grüner, Bethesda Spital, Stephan Harbarth, Hôpitaux Universitaires de Genève HUG, Ulrich Heininger, Universitäts-Kinderspital beider Basel UKBB, Margret Hund-Georgiadis, REHAB Basel, Jörg Isenegger, Spital Netz Bern AG Spital Riggisberg, Andreas Jenny, Schweizer Paraplegiker-Zentrum, Chloé Kaech, St. Claraspital AG, Markus Klink, Bürgerspital Basel Reha Chrischona, Stefan Kuster, Universitätsspital Zürich, Reto Laffer, SRO AG Spital Langenthal, Jonas Marschall, Inselspital Universitätsspital Bern, Anita Niederer-Loher, Ostschweizer Kinderspital, Reto Nüesch, Spital Schwyz, Christina Orasch, Hirslanden Klinik St. Anna, Jan Rein Piso, Solothurner Spitäler AG (soH) Kantonsspital Olten und Spital Dornach und Bürgerspital Solothurn, Rolf Prions, Gesundheitszentrum Fricktal AG Spital Rheinfelden, Philippe Rafeiner, Spital Zofingen AG, Ferdinand Repond, Spital Netz Bern AG Spital Münsingen und Spital Ziegler, Christiane Resch, Spital STS AG Spital Zweisimmen, Marco Rossi, Luzerner Kantonsspital LUKS Luzern und Wolhusen und Sursee, Hugo Sax, Universitätsspital Zürich, Matthias Schlegel, Kantonsspital St. Gallen und Spitalregion Rheintal Werdenberg Sarganserland, Hervé Schlotterbeck, Fondazione Cardiocentro Ticino,

Alexander Spillmann, Kreisspital für das Freiamt Muri, Philip Tarr, Kantonsspital Baselland Bruderholz, Nicolas Troillet, Hôpital du Valais (RSV) Centre Hospitalier du Valais Romand (CHVR) und Spitalzentrum Oberwallis (SZO), Reto Weber, fmi-Spitäler Spital Frutigen, Georgio Zanetti, Centre hospitalier universitaire vaudois CHUV, Etablissements Hospitaliers du Nord Vaudois eHnv – Site de St-Loup, Hôpital du Chablais Site d'Aigle, Hôpital du Chablais Site de Monthey, Hôpital fribourgeois HFR Site de Riaz, Kantonsspital Frauenfeld und Kantonsspital Münsterlingen, Spital Altstätten und Spital Walenstadt, Spital Grabs, Spital Heiden und Herisau,Spital Flawil und Spital Rorschach, Spital Linth, Spital Thurgau AG, Spitalregion Fürstenland Toggenburg, Spitalverbund Appenzell Ausserrhoden, Spital Wattwil und Spital Will,

We thank all laboratories and local coordinators (in alphabetical order): Christoph Berger, Universitäts-Kinderkliniken Zürich Infektionslabor, Luce Bertaiola Monnerat, HFR Laboratoire de microbiologie, Thomas Bodmer, Labormedizinisches Zentrum Dr. Risch AG / Liebefeld BE, Dieter Burki, Luzerner Kantonsspital Institut für Medizinische Mikrobiologie, Selja Capaul, medica MEDIZINISCHE LABORATORIEN Dr. F. KAEPPELI AG, Sara Droz, Universität Bern Institut für Infektionskrankheiten Klinische Mikrobiologie, Olivier Dubius, Viollier AG Allschwil, Adrian Egli, Universitätsspital Basel Labormedizin Klinische Mikrobiologie, Gerhard Eich, Stadtspital Triemli Institut für Labormedizin Bakteriologie-Labor, Andrea Elio d' Andrea, Ospedale San Sisto Labor Team W, Barbara Erb, Spitalzentrum Biel Zentrallabor, Hans Fankhauser, Institut für Labormedizin Aarau, Susanne Graf, KS Baselland, Liestal Zentrallaboratorien Bakteriologielabor und Bruderholz Zentrallaboratorien Bakteriologielabor, Gilbert Greub, CHUV / Institut de microbiologie Département des laboratoires, Irene Gwerder, Spital Schwyz Mikrobiologie Labor, Kathrin Herzog, Kantonsspital Frauenfeld Institut für Labormedizin / Mikrobiologielabor und Kantonsspital Münsterlingen Institut für Labormedizin / Mikrobiologielabor, Hans H. Hirsch, IMM Abteilung für Infektionsdiagnostik Department Biomedizin Basel – Haus Petersplatz, Alexander Imhof, SRO AG, Nadia Liassine, Dianalabs, Gladys Martinelli, Servicio di Microbiologia EOLAB, Ursula Notter Gut, Spital Davos Labor, Sigrid Pranghofer, Bioanalytica AG, Gérard Praz, Institut Central ICHV, Susanne Rieser Schöni, Schweizer Paraplegiker Zentrum Labor, Martin Risch, KS Graubünden Zentrallabor Chur ZLC und Labormedizinisches Zentrum Dr. Risch / Schaan, Urs Schibli, Bakteriologisches Institut Olten (BIO), Jacques Schrenzel, HUG Laboratoire de bactériologie, Detlev Schultze, Zentrum für Labormedizin SG, Hans H. Siegrist, ADMED Microbiologie, Spital Langenthal, Angelika Ströhle, mcl / Medizinische Laboratorien, Sabrina Trachsel, eHnv Laboratoire de microbiologie site Yverdon, Reinhard Zbinden, Universität Zürich Institut für medizinische Mikrobiologie.
